# Socioeconomic gradients in diabetes prevalence across Israeli municipalities: the role of population density

**DOI:** 10.3389/fepid.2026.1848489

**Published:** 2026-10-19

**Authors:** Yuval Arbel, Israel Rachevski, Amichai Kerner, Miryam Kerner

**Affiliations:** 1 Sir Harry Solomon School of Economics and Management, Western Galilee College, Acre, Israel; 2 Sir Harry Solomon School of Economics and Management, Western Galilee College, Acre, Israel; 3 Faculty of Social Sciences, Banking and Finance Program, Bar Ilan University, Ramat Gan, Israel; 4 The Ruth and Bruce Rapoport Faculty of Medicine, Technion – Israel Institute of Technology, Haifa, Israel; 5 Department of Dermatology, Kaplan Medical Center, Rehovot, Israel

**Keywords:** diabetes prevalence, health inequalities, health policy, population density, socioeconomic status

## Abstract

**Background:**

Health policies often prioritize the most socioeconomically disadvantaged populations, implicitly assuming that health outcomes improve monotonically with socioeconomic status. However, this assumption may obscure important patterns in disease distribution and lead to suboptimal targeting of interventions.

**Methods:**

This study uses municipality-level data covering 127 Israeli municipalities to examine the relationship between municipal socioeconomic rank and diabetes prevalence and whether this relationship varies with population density. A quadratic specification interacted with population density is used to allow for non-linear gradients in health outcomes across different levels of density.

**Results:**

Diabetes prevalence is strongly negatively associated with socioeconomic rank at the municipal level, but the relationship between population density and diabetes prevalence varies across the socioeconomic distribution. Higher population density is associated with significantly lower diabetes prevalence among municipalities at SES ranks 1–3, whereas this association is not statistically significant at higher SES ranks. The quadratic SES component also becomes more negative as population density increases and is statistically significant at the 75th-percentile density, although this evidence of density-dependent curvature should be interpreted cautiously.

**Conclusions:**

These findings challenge the common assumption of a uniform socioeconomic gradient in health across different urban contexts. In the Israeli context, policies that focus primarily on socioeconomic status without considering population density may overlook important differences in disease burden. Incorporating interactions between socioeconomic gradients and population density, together with outcome-based indicators, may provide useful additional information for identifying heterogeneity in diabetes burden and informing the targeting of prevention strategies.

## 1 Introduction

Reducing health inequalities is a central objective of public health policy. A substantial body of research shows that health outcomes are shaped not only by individual characteristics but also by place-based factors such as local environments, access to services, and institutional capacity (1–3). These place effects are highly relevant for policymakers, as health systems often allocate resources geographically, targeting populations based on area-level socioeconomic conditions. In parallel, insights from urban and regional economics highlight how agglomeration forces, amenities, and spatial equilibrium generate persistent territorial disparities (4–7), further reinforcing the importance of spatially informed policy design.

A common implicit assumption underlying many policy frameworks is that socioeconomic gradients in health are monotonic—that is, health outcomes improve steadily as socioeconomic conditions improve. This assumption underpins widely used targeting strategies that prioritize the most disadvantaged populations and areas. Empirical evidence generally supports the existence of worse health outcomes among lower socioeconomic groups (8–10). However, while analytically convenient, the monotonicity assumption may obscure more complex patterns in the distribution of disease burden.

Emerging evidence suggests that the relationship between socioeconomic conditions, the built environment, and health may be non-linear, reflecting transitions in consumption patterns, mobility, and institutional capacity (11–13). Moreover, these socioeconomic gradients may vary with characteristics of the urban environment, including population density. From a policy perspective, such non-linearities and interactions are critical: if disease risk does not decline uniformly with socioeconomic status across different urban contexts, then standard targeting approaches may fail to identify populations at greatest risk.

Municipalities constitute key units for health policy implementation, as they combine income levels, infrastructure, service provision, and environmental characteristics. Movement along the socioeconomic hierarchy of municipalities may involve structural transitions that do not translate into uniform improvements in health outcomes. Population density may be particularly relevant in this context, as denser municipalities differ in accessibility, infrastructure, service provision, mobility patterns, and the concentration of health-related resources and exposures. Consequently, the association between socioeconomic conditions and health outcomes may vary across different levels of population density.

This paper examines whether the municipal socioeconomic gradient in diabetes prevalence varies across population-density contexts and, in particular, whether the association between population density and diabetes prevalence differs across the socioeconomic distribution. Using municipality-level administrative data for 127 Israeli municipalities, we identify a strong negative association between municipal socioeconomic rank and diabetes prevalence, together with systematic variation in the shape of this relationship across population-density levels. In particular, significant negative curvature in the socioeconomic gradient emerges at relatively high population densities, while higher density is associated with lower diabetes prevalence among municipalities at the lowest socioeconomic ranks.

The findings have direct implications for public health policy. If socioeconomic gradients in health vary across population-density contexts, policies that focus primarily on socioeconomic rank alone may misallocate resources and overlook important differences in disease burden across municipalities. Considering socioeconomic gradients together with population density and other contextual characteristics may therefore provide additional information for identifying heterogeneity in disease burden and informing prevention and resource-allocation strategies.

This study contributes to the literature in three ways. First, it provides evidence that socioeconomic gradients in health outcomes at the municipal level may vary with population density. Second, it demonstrates that commonly used monotonic specifications may misrepresent the distribution of disease burden when interactions between socioeconomic conditions and the urban environment are ignored. Third, and most importantly from a policy perspective, it shows that place-based targeting strategies may benefit from considering both socioeconomic conditions and population density rather than relying on socioeconomic rank alone.

## 2 Related literature

This paper relates to four strands of literature that inform both the understanding of health inequalities and the design of effective health policy.

### 2.1 Spatial inequality and regional economics

Foundational work highlights how agglomeration, amenities, and spatial equilibrium generate uneven outcomes across places (4–7, 14, 15). Empirical work often assumes monotonic gradients. We contribute by showing that municipal health gradients may vary with population density.

### 2.2 Health geography and place effects

Health outcomes reflect neighborhood context, environmental exposures, and access to services (1–3, 16). Municipal SES indices bundle multiple place-based attributes, making them natural proxies for territorial conditions affecting health.

### 2.3 Socioeconomic gradients in health

A large literature documents worse outcomes in disadvantaged populations and places (8–10, 17, 18). Related work links food environments and access to obesity and diabetes (12, 19–21). These studies typically assume monotonicity.

### 2.4 Heterogeneity across population-density contexts

Emerging evidence indicates non-linear effects of density, built environment, and socioeconomic context (11, 13). Such effects suggest that socioeconomic health gradients may differ across population-density contexts rather than following a uniform pattern across municipalities.

Overall, we contribute by documenting a strong negative municipal socioeconomic gradient in diabetes prevalence while showing that the association between population density and diabetes prevalence is concentrated primarily among socioeconomically disadvantaged municipalities. We additionally examine whether the shape of the SES gradient varies across population-density contexts.

## 3 Descriptive statistics

The analytical sample includes 127 of the 132 municipalities in the full dataset, corresponding to approximately 96% coverage. The sample includes all municipalities for which complete information was available on diabetes prevalence, socioeconomic rank, and population density. The remaining five municipalities were excluded because information on one or more of these variables was unavailable. Thus, the analytical sample provides broad geographic coverage across Israel, although it does not include the complete universe of municipalities represented in the dataset.

The dependent variable is age-standardized diabetes prevalence, measured as the age-standardized number of diagnosed diabetes cases per 1,000 inhabitants at the municipal level, based on data for 2017–2019.

Socioeconomic status is measured using the official municipal socioeconomic rank published by the Israeli Central Bureau of Statistics. The official SES scale ranges from 1 (lowest) to 10 (highest); in the present analytical sample, observed ranks range from 1 to 9. The socioeconomic ranking reflects multiple dimensions of local socioeconomic conditions, including income, education, employment, and living conditions.

Population density, measured as inhabitants per square kilometer, is incorporated to capture differences in the urban context across municipalities. Because the density distribution is right-skewed, the empirical analysis uses the natural logarithm of population density. The interaction model presented below centers ln(Density) at the sample median density of 2,713.2 inhabitants per km^2^.

Figure 1 illustrates the distribution of diabetes prevalence across socioeconomic ranks in the 127 municipalities. Each point represents one municipality, while diamonds indicate mean diabetes prevalence within each socioeconomic rank. The figure shows substantial variation in diabetes prevalence both across and within socioeconomic ranks. Although diabetes prevalence generally declines as socioeconomic rank increases, considerable within-rank dispersion remains, suggesting that socioeconomic rank alone does not fully characterize differences in municipal diabetes prevalence.

**Figure 1:**
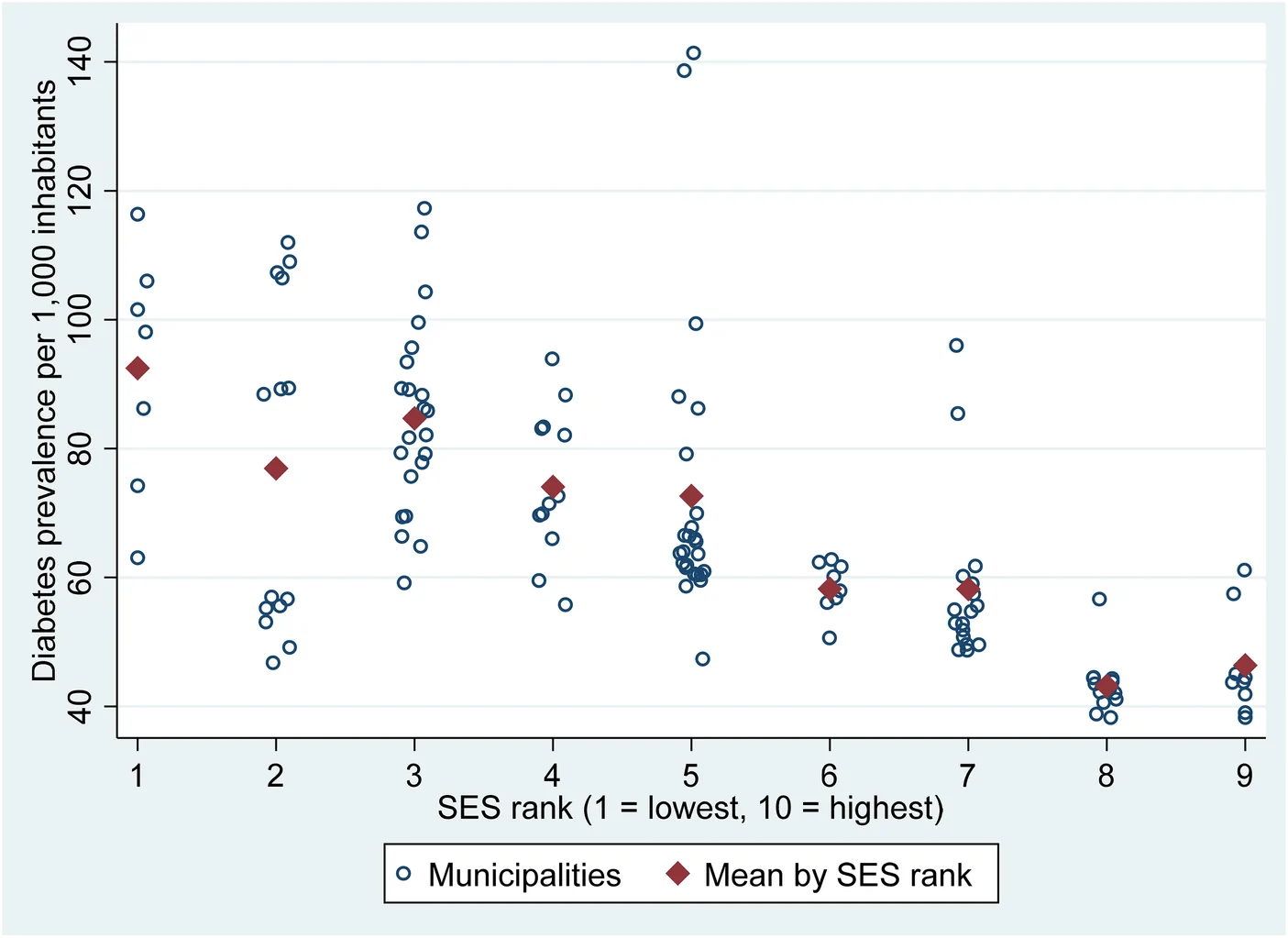
Observed diabetes prevalence across socioeconomic ranks. Each point represents one municipality in the analytical sample (*N* = 127). Diabetes prevalence is measured as cases per 1,000 inhabitants. The official SES scale ranges from 1 (lowest) to 10 (highest); observed ranks in the analytical sample range from 1 to 9. Points are horizontally jittered solely to distinguish municipalities sharing the same SES rank. Diamonds indicate the mean diabetes prevalence within each SES rank.

Because socioeconomic rank is an ordinal measure with nine observed levels in the analytical sample, the quadratic specification should be interpreted as a parsimonious representation of potential curvature rather than as uniquely identifying the functional form of the SES–diabetes relationship. Accordingly, the regression results are interpreted alongside the rank-specific observed patterns shown in Figure 1.

Table 1 presents descriptive statistics for the variables included in the analysis. The mean diabetes prevalence is 68.71 cases per 1,000 inhabitants (SD = 21.79), with values ranging from 38.27 to 142.84. Municipal socioeconomic rank has a mean of 4.93 (SD = 2.31) and ranges from 1 to 9. Mean population density is 3,933.92 inhabitants per km^2^, compared with a median of 2,713.20, reflecting the right-skewed density distribution. The municipalities also vary substantially in population size, with a mean population of 62,249 and a median of 28,753.

**Table 1:** Descriptive statistics for the municipality-level sample.

Variable	*N*	Mean	Std. dev.	Min	Median	Max
Diabetes prevalence (per 1,000 inhabitants)	127	68.71	21.79	38.27	62.17	142.84
SES rank	127	4.93	2.31	1	5	9
Population	127	62,249	107,457	6,738	28,753	981,711
Population density (inhabitants/km^2^)	127	3,933.92	3,889.65	166.80	2,713.20	29,714.40
ln(Population density)	127	7.960	0.807	5.117	7.906	10.299

The unit of observation is the municipality (*N* = 127). Diabetes prevalence is measured as diagnosed diabetes cases per 1,000 inhabitants. The official SES scale ranges from 1 (lowest) to 10 (highest); observed ranks in the analytical sample range from 1 to 9. Population density is measured as inhabitants per square kilometer. The median population density is 2,713.2 inhabitants/km^2^; its natural logarithm is used as the centering value in the interaction model reported in Table 3.

Table 2 reports Pearson correlation coefficients between the key variables. Diabetes prevalence is strongly negatively correlated with municipal socioeconomic rank (r=−0.633,p<0.001) and is also negatively correlated with ln(Density) (r=−0.273,p=0.002) and population (r=−0.244,p=0.006). SES rank is positively correlated with ln(Density) (r=0.219,p=0.014), while its correlation with population is small and statistically insignificant (r=0.066,p=0.464). These bivariate relationships provide descriptive evidence of socioeconomic and density-related differences in diabetes prevalence, while the multivariable analysis below examines whether the socioeconomic gradient itself varies with population density.

**Table 2:** Pearson correlations among municipality-level variables.

Variable	(1) Diabetes prevalence	(2) SES rank	(3) ln(Density)	(4) Population
(1) Diabetes prevalence	1.000			
(2) SES rank	−0.633***	1.000		
(3) ln(Density)	−0.273***	0.219**	1.000	
(4) Population	−0.244***	0.066	0.348***	1.000

Pearson correlation coefficients are reported for the 127 municipalities in the analytical sample. Diabetes prevalence is measured as diagnosed diabetes cases per 1,000 inhabitants. The official SES scale ranges from 1 (lowest) to 10 (highest); observed ranks in the analytical sample range from 1 to 9. Population density is expressed in natural logarithms.

*** *p* < 0.01.

** *p* < 0.05.

## 4 Methods

The empirical analysis examines whether the association between municipal socioeconomic status and diabetes prevalence varies with population density. Because both diabetes prevalence and socioeconomic rank are measured at the municipality level, the municipality is treated as the unit of observation. The analytical sample consists of 127 municipalities with non-missing information on diabetes prevalence, socioeconomic rank, and population density.

To allow for potential non-linearity in the socioeconomic gradient, the model includes both the linear and squared terms of municipal socioeconomic rank. Population density is entered in natural logarithms because of its strongly right-skewed distribution. To facilitate interpretation of the interaction terms, ln(Density) is centered at the sample median, corresponding to approximately 2,713 inhabitants per km^2^.

The empirical model is specified as:

$$\begin{array}{rl} \mathrm{Diabete}s_{i}= & \beta_{0}+\beta_{1}\mathrm{SE}S_{i}+\beta_{2}{\mathrm{SES}}_{i}^{2}+\beta_{3}c\ln(\mathrm{Densit}y_{i})\\ & +\beta_{4}[\mathrm{SE}S_{i}\times c\ln(\mathrm{Densit}y_{i})]\\ & +\beta_{5}[{\mathrm{SES}}_{i}^{2}\times c\ln(\mathrm{Densit}y_{i})]+\varepsilon_{i} \end{array}$$

where $\mathrm{Diabete}s_{i}$ denotes the number of diagnosed diabetes cases per 1,000 inhabitants in municipality *i*, $\mathrm{SE}S_{i}$ is the official municipal socioeconomic rank, and $c\ln(\mathrm{Densit}y_{i})$ is the natural logarithm of population density centered at the sample median. The interaction terms allow both the slope and curvature of the SES–diabetes relationship to vary with population density.

The model is estimated using ordinary least squares (OLS) with HC3 heteroskedasticity-robust standard errors. HC3 standard errors are used to provide inference that is less sensitive to heteroskedasticity and influential observations in a relatively small cross-sectional sample.

The conditional effect of population density at a given SES rank *s* is:

$$\frac{\partial \mathrm{Diabetes}}{\partial \ln(\mathrm{Density})}=\beta_{3}+\beta_{4}s+\beta_{5}s^{2}.$$

This expression is used to calculate the marginal effect of population density at SES ranks 1–9. For ease of interpretation, the estimated semi-elasticities are also converted into the predicted change in diabetes prevalence associated with an exact 10% increase in density by multiplying the conditional effect by ln(1.10).

Conversely, the conditional SES relationship at a given population density *D* is characterized by:

$$[\beta_{1}+z\beta_{4}]SES+[\beta_{2}+z\beta_{5}]SES^{2},$$

where

z=ln(D)−ln(2713.2).

This formulation allows the linear and quadratic components of the SES gradient to be evaluated at different population-density levels. Conditional coefficients are estimated at the 25th-percentile density (1,927 inhabitants/km^2^), the median density (2,713 inhabitants/km^2^), and the 75th-percentile density (4,531 inhabitants/km^2^). The statistical significance of the conditional quadratic term is evaluated using linear combinations of the estimated coefficients and the HC3 covariance matrix.

In addition, the approximate density region in which the conditional quadratic SES coefficient becomes statistically significant at the 5% level is calculated from the estimated coefficient vector and HC3 covariance matrix. This provides an indication of the population-density region in which statistically detectable curvature in the SES–diabetes relationship emerges and should not be interpreted as identifying a discrete substantive threshold.

## 5 Results

The municipality-level analysis indicates that the relationship between socioeconomic status and diabetes prevalence varies systematically with population density. Rather than identifying a uniform non-monotonic socioeconomic gradient across municipalities, the results show that both the slope and curvature of the SES–diabetes relationship depend on the density context. In particular, population density is associated with lower diabetes prevalence primarily among municipalities at the lowest socioeconomic ranks, while statistically significant curvature in the SES gradient emerges only at relatively high population densities.

### 5.1 Main regression results

Table 3 presents the estimates from the interaction model in which diabetes prevalence is regressed on SES rank, SES rank squared, centered ln(Density), and the interactions of centered ln(Density) with both SES and SES^2^.

**Table 3:** Population density as a moderator of the nonlinear association between socioeconomic status and diabetes prevalence dependent variable: diabetes prevalence per 1,000 inhabitants.

Variable	Coefficient	HC3 Robust SE	*p*-value
Constant	89.526***	7.830	<0.001
SES rank	−2.094	3.279	0.523
SES rank^2^	−0.358	0.301	0.233
Centered ln(Density)	−20.861***	8.088	0.010
SES × centered ln(Density)	6.652**	3.147	0.035
SES^2^ × centered ln(Density)	−0.526*	0.280	0.060
Observations	127		
*R* ^2^	0.442		
Adjusted *R*^2^	0.419		

HC3 heteroskedasticity-robust standard errors are reported. ln(Density) is centered at the sample median, corresponding to approximately 2,713 inhabitants/km^2^. The official SES scale ranges from 1 (lowest) to 10 (highest); observed ranks in the analytical sample range from 1 to 9. Consequently, the coefficients on SES rank and SES rank^2^ represent the SES relationship at the median population density.

*** *p* < 0.01.

** *p* < 0.05.

* *p* < 0.10.

At the median population density, corresponding to approximately 2,713 inhabitants/km^2^, neither the linear SES coefficient nor the quadratic SES coefficient is statistically significant. The coefficient on SES rank is −2.094 (p=0.523), while the coefficient on SES^2^ is −0.358 (p=0.233). These coefficients should therefore not be interpreted as evidence of a general nonlinear SES gradient at the median density.

Population density, however, significantly modifies the socioeconomic gradient. The coefficient on centered ln(Density) is negative and highly statistically significant (−20.861, p=0.010). In addition, the interaction between SES rank and centered ln(Density) is positive and statistically significant (6.652, p=0.035), while the interaction between SES^2^ and centered ln(Density) is negative and marginally significant (−0.526, p=0.060). Taken together, these interaction terms indicate that the association between population density and diabetes prevalence changes across the socioeconomic distribution and that the curvature of the SES gradient becomes more negative as population density increases.

The model is estimated using 127 municipalities and explains approximately 44.2% of the cross-sectional variation in diabetes prevalence ($R^{2}=0.442$; adjusted $R^{2}=0.419$).

### 5.2 Predicted SES gradients across population-density levels

Figure 2 illustrates the implications of the interaction model by plotting predicted diabetes prevalence across SES ranks at three representative population densities: the 25th percentile (1,927 inhabitants/km^2^), the median (2,713 inhabitants/km^2^), and the 75th percentile (4,531 inhabitants/km^2^).

**Figure 2:**
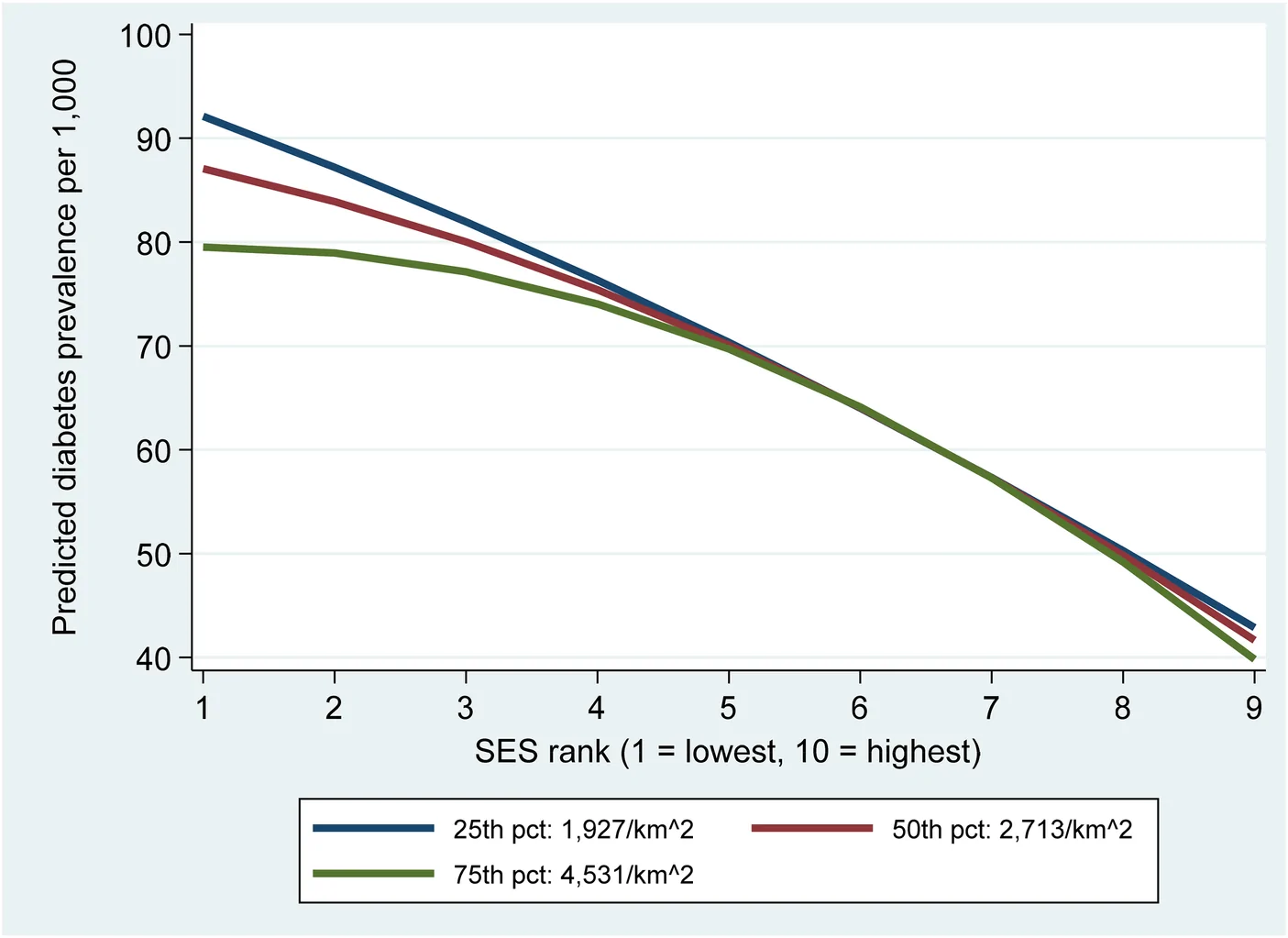
Predicted diabetes prevalence by socioeconomic rank at different levels of population density. Predicted values are based on the estimates reported in Table 3. Curves are evaluated at the 25th percentile (1,927 inhabitants/km^2^), median (2,713 inhabitants/km^2^), and 75th percentile (4,531 inhabitants/km^2^) of the population-density distribution. The official SES scale ranges from 1 (lowest) to 10 (highest); observed ranks in the analytical sample range from 1 to 9.

The figure demonstrates that the SES–diabetes relationship is not constant across density levels. At relatively low population density, the predicted SES gradient is comparatively weak. As population density increases, the predicted relationship becomes increasingly curved and the differences between low- and high-SES municipalities become more pronounced.

The three curves are especially separated at the lower end of the SES distribution. At low SES ranks, predicted diabetes prevalence is substantially lower in denser municipalities than in municipalities with lower population density. This density-related difference diminishes as socioeconomic rank increases, and the predicted curves converge around the middle and upper portions of the SES distribution.

Thus, Figure 2 suggests that population density is particularly relevant to diabetes prevalence among socioeconomically disadvantaged municipalities, while its association is considerably weaker at higher SES levels.

### 5.3 Marginal effect of population density across socioeconomic ranks

Table 4 quantifies the pattern shown in Figure 2 by reporting the marginal effect of ln(Density) at each SES rank.

**Table 4:** Marginal effect of population density on diabetes prevalence by socioeconomic rank.

SES rank	Marginal effect of ln(Density)	*p*-value	Effect of a 10% increase in density
1	**−14.73**	**0.006**	**−1.40**
2	**−9.66**	**0.005**	**−0.92**
3	**−5.64**	**0.012**	**−0.54**
4	−2.66	0.165	−0.25
5	−0.74	0.707	−0.07
6	+0.13	0.947	+0.01
7	−0.05	0.976	−0.01
8	−1.29	0.530	−0.12
9	−3.57	0.269	−0.34

Marginal effects are calculated from the interaction model reported in Table 3. At SES rank *s*, the marginal effect of ln(Density) is $\beta_{\ln\;\mathrm{Density}}+s\beta_{\mathrm{SES}\times \ln\;\mathrm{Density}}+s^{2}\beta_{\mathrm{SE}S^{2}\;\times \ln\;\mathrm{Density}}$. The final column converts this semi-elasticity into the predicted change in diabetes prevalence associated with an exact 10% increase in population density, calculated by multiplying the marginal effect by ln(1.10). Effects are expressed as diabetes cases per 1,000 inhabitants. *P*-values are based on HC3 heteroskedasticity-robust standard errors.

Bold values indicate statistical significance at the 5% level (*p* < 0.05).

At SES rank 1, the marginal effect of ln(Density) is −14.73 (p=0.006). An exact 10% increase in population density is therefore associated with approximately 1.40 fewer diabetes cases per 1,000 inhabitants. At SES rank 2, the corresponding effect is −9.66 (p=0.005), equivalent to approximately 0.92 fewer cases per 1,000 for a 10% increase in density. At SES rank 3, the marginal effect remains negative and statistically significant (−5.64, p=0.012), corresponding to approximately 0.54 fewer diabetes cases per 1,000 inhabitants for a 10% increase in density.

Beginning at SES rank 4, the marginal effect of population density is no longer statistically distinguishable from zero. The estimated effect declines to −2.66 at SES rank 4 (p=0.165) and remains statistically insignificant throughout SES ranks 5–9.

These results provide a more precise interpretation of the density interaction: higher population density is associated with lower diabetes prevalence primarily among municipalities in the lowest socioeconomic ranks. The apparent density-related advantage diminishes progressively as SES increases and is no longer statistically significant from approximately SES rank 4 onward.

### 5.4 Conditional SES gradient at different population densities

Table 5 examines the interaction from the complementary perspective by estimating the conditional linear and quadratic SES coefficients at the same three density levels used in Figure 2.

**Table 5:** Conditional socioeconomic gradient in diabetes prevalence at selected population densities.

Population density (inhabitants/km^2^)	Conditional SES coefficient	*p*-value	Conditional SES^2^ coefficient	*p*-value
1,927 (25th percentile)	−4.370	0.230	−0.178	0.595
2,713 (median)	−2.094	0.523	−0.358	0.236
4,531 (75th percentile)	+1.317	0.699	**−0.628**	**0.040**

Conditional coefficients are linear combinations of the coefficients reported in Table 3 and are evaluated at the indicated population densities. For density *D*, the conditional linear SES coefficient is $\beta_{\mathrm{SES}}+[\ln(D)-\ln(2,713.2)]\beta_{\mathrm{SES}\times \ln\;\mathrm{Density}}$, and the conditional quadratic coefficient is $\beta_{\mathrm{SE}S^{2}}+[\ln(D)-\ln(2,713.2)]\beta_{\mathrm{SE}S^{2}\;\times \ln\;\mathrm{Density}}$. *p*-values are based on HC3 heteroskedasticity-robust standard errors. The conditional quadratic coefficient becomes statistically significant at the 5% level at a population density of approximately 4,227 inhabitants/km^2^. At the 75th-percentile density (4,531 inhabitants/km^2^), the quadratic component is negative and statistically significant, indicating significant downward curvature in the SES–diabetes relationship. The estimated turning point at this density is approximately SES rank 1.05. Ths value should be interpreted as indicating an approximate density region in which curvature becomes statistically detectable rather than as a discrete substantive threshold.

Bold values indicate statistical significance at the 5% level (*p* < 0.05).

At the 25th-percentile density of 1,927 inhabitants/km^2^, the conditional quadratic SES coefficient is −0.178 and is not statistically significant (p=0.595). At the median density of 2,713 inhabitants/km^2^, the coefficient is −0.358 and also remains statistically insignificant (p≈0.236). In contrast, at the 75th-percentile density of 4,531 inhabitants/km^2^, the conditional quadratic coefficient becomes substantially more negative (−0.628) and statistically significant at the 5% level (p=0.040).

The analysis also identifies the approximate density region in which the conditional quadratic SES coefficient becomes statistically detectable at the 5% level. This occurs at approximately 4,227 inhabitants/km^2^. This value should not be interpreted as a discrete substantive threshold, but rather as an approximate region in which downward curvature becomes statistically detectable.

At the 75th-percentile density, the conditional quadratic coefficient is negative and statistically significant, indicating significant downward curvature in the SES–diabetes relationship. To assess the substantive meaning of this curvature, I calculated the implied turning point and examined its location relative to the observed SES distribution. The maximum is given by $\mathrm{SE}S^{*}=-b/(2a)$, where *b* and *a* are the conditional linear and quadratic SES coefficients, respectively. Using the estimated coefficients at the 75th-percentile density (b=1.317anda=−0.628) gives a maximum at approximately SES rank 1.05. Because this implied turning point lies almost exactly at the lower boundary of the observed SES distribution (SES rank 1), the result should not be interpreted as evidence of a broad inverted-U relationship with a peak at intermediate socioeconomic levels. Rather, predicted diabetes prevalence reaches its maximum near the bottom of the observed SES range and declines across virtually the entire remaining SES distribution.

### 5.5 Summary of empirical findings

Taken together, the results provide evidence that:

- the socioeconomic gradient in diabetes prevalence is conditional on population density, rather than following a single uniform functional form across municipalities;
- greater population density is associated with significantly lower diabetes prevalence at SES ranks 1–3, but this association is not statistically significant at higher SES ranks;
- the curvature of the SES–diabetes relationship becomes increasingly negative as population density rises;
- statistically significant downward curvature emerges at densities above approximately 4,227 inhabitants/km^2^; and
- the high-density relationship should not be characterized as a broad inverted-U pattern, because its estimated turning point lies near the lower boundary of the SES distribution.

Overall, the findings suggest that socioeconomic conditions and population density should be considered jointly when examining geographic variation in diabetes prevalence. A model based solely on a uniform socioeconomic gradient would obscure meaningful differences between low- and high-density municipalities.

## 6 Discussion

### 6.1 Interpretation of findings

This study identifies a density-dependent relationship between municipal socioeconomic rank and diabetes prevalence. Rather than exhibiting a uniform socioeconomic gradient across municipalities, the relationship varies systematically with population density. This finding challenges the assumption that the association between socioeconomic conditions and health can be adequately characterized by a single functional form across different urban contexts and suggests a more complex distribution of health risks across municipalities.

The results reveal two complementary patterns. First, higher population density is associated with significantly lower diabetes prevalence among municipalities at the lowest socioeconomic ranks, with the largest estimated density-related difference occurring at SES rank 1. This association becomes progressively weaker as socioeconomic rank increases and is no longer statistically significant from SES rank 4 onward. Second, the curvature of the SES–diabetes relationship becomes increasingly negative as population density rises. At relatively high densities, the quadratic component of the socioeconomic gradient becomes statistically significant, indicating downward curvature in the relationship between SES and diabetes prevalence.

One possible interpretation is that population density may capture differences in the local environments in which socioeconomic conditions translate into health outcomes. Denser municipalities may provide greater proximity to services, infrastructure, and other resources that are particularly relevant to disadvantaged populations. At the same time, socioeconomic conditions may interact with behavioral and environmental characteristics associated with urban form. Consumption patterns, physical activity, and access to health-supportive resources have been linked to socioeconomic and built-environment conditions in the existing literature (12, 20). These mechanisms could contribute to differences in the socioeconomic gradient across population-density contexts, although they cannot be directly identified using the present data. Population density should therefore not be interpreted as the causal mechanism underlying these differences. Rather, it may proxy for a broader set of municipal characteristics, including urbanicity, demographic composition, transportation accessibility, healthcare infrastructure, regional location, and service availability.

This interpretation is consistent with a place-based perspective in which health outcomes emerge from the interaction between socioeconomic conditions, local environments, and service provision (1–3). It also aligns with emerging evidence on non-linear effects of the built environment and socioeconomic context (11, 13). Importantly, the results suggest that the relationship between socioeconomic status and health is not uniform across space, but instead may depend on characteristics of the local environment, including population density.

### 6.2 Policy implications

The findings have direct implications for health policy design and resource allocation. Many health systems, including in Israel, allocate preventive resources and interventions based on socioeconomic ranking, often prioritizing the most disadvantaged municipalities. While this approach is well justified, it may implicitly assume that the relationship between socioeconomic disadvantage and health risk is similar across different municipal contexts.

The results of this study suggest that socioeconomic rank alone may not fully characterize differences in diabetes prevalence across municipalities. Population density modifies the association between socioeconomic status and diabetes prevalence, with particularly pronounced density-related differences among municipalities at the lowest socioeconomic ranks. Consequently, municipalities with similar socioeconomic rankings may exhibit different health outcomes depending on their population-density context.

Incorporating local contextual characteristics alongside socioeconomic gradients into policy frameworks may provide additional information for identifying heterogeneity in disease burden and informing targeting decisions. In practice, this may involve:

- complementing socioeconomic indices with direct health outcome indicators (e.g., disease prevalence);
- identifying municipalities with unexpectedly high disease burden relative to their socioeconomic rank and local characteristics; and
- considering population density and other characteristics of the local environment when designing screening, health-promotion, and lifestyle interventions.

More broadly, the findings suggest that policy frameworks should move beyond simple socioeconomic rankings and adopt more flexible approaches that account for heterogeneity in local health risks. Such approaches are particularly relevant for chronic disease prevention, where risk factors are closely linked to environmental and behavioral conditions and where the association between socioeconomic conditions and health may differ substantially across places.

### 6.3 Limitations

Several limitations should be considered when interpreting the results. First, the analysis covers 127 of the 132 municipalities in the full dataset, corresponding to approximately 96% coverage. The five municipalities for which complete information on diabetes prevalence, socioeconomic rank, and population density was unavailable were excluded from the analytical sample. Thus, although the analysis provides broad geographic coverage across Israel, it does not include the complete universe of municipalities represented in the dataset, and some degree of selection associated with missing data cannot be excluded.

Second, the empirical approach is observational and does not establish causal relationships. The estimated associations between socioeconomic rank, population density, and diabetes prevalence may reflect behavioral, environmental, demographic, and institutional factors that cannot be fully disentangled in the current framework. Although the diabetes prevalence measure is age-standardized, residual confounding by other unobserved demographic and municipality-level characteristics cannot be excluded. Population density in particular should therefore be interpreted as a contextual characteristic rather than as an exogenous treatment. Future research should explore these mechanisms more directly, potentially using longitudinal data or quasi-experimental designs.

Third, the analysis relies on municipality-level data. Accordingly, the estimated relationships describe differences between municipalities and should not be interpreted as individual-level associations between socioeconomic status, residential density, and diabetes risk. Within-municipality heterogeneity in socioeconomic conditions and health outcomes cannot be observed in the present data.

Finally, the study focuses on diabetes prevalence as a key indicator of population health. While diabetes is highly relevant for public health policy, examining additional outcomes could provide a more comprehensive understanding of how socioeconomic gradients in health vary across different population-density contexts.

## 7 Conclusions

This study documents systematic variation in the socioeconomic gradient of diabetes prevalence across population-density contexts. Rather than supporting a single uniform relationship between municipal socioeconomic rank and diabetes prevalence, the results indicate that population density modifies both the magnitude and shape of this association.

Two findings are particularly noteworthy. First, higher population density is associated with significantly lower diabetes prevalence among municipalities at the lowest socioeconomic ranks, with the largest estimated density-related difference occurring at SES rank 1. This association becomes weaker and statistically insignificant at higher SES ranks. Second, there is more modest evidence that the shape of the SES–diabetes relationship also varies with population density, with the quadratic component becoming statistically significant at the 75th-percentile density. These findings indicate that socioeconomic and urban characteristics should be considered jointly when examining geographic inequalities in diabetes.

From a policy perspective, the results suggest that health systems that allocate resources primarily on the basis of socioeconomic disadvantage may benefit from incorporating additional information on local context and observed health outcomes. Municipalities with similar socioeconomic rankings may face different disease burdens depending on their population-density environment.

Population density and other local contextual characteristics may provide useful additional information for understanding heterogeneity in disease burden and informing resource-allocation and targeting decisions. Complementing socioeconomic indicators with direct measures of health outcomes may also help identify municipalities with comparatively high observed disease burden and inform the deployment of preventive services.

More broadly, the findings highlight the need to reconsider simplified assumptions about the relationship between socioeconomic status and health. Socioeconomic gradients may vary across different urban contexts rather than operating uniformly across municipalities. Recognizing this heterogeneity can contribute to more nuanced place-based public health policies, particularly in the context of chronic disease prevention.

Future research should examine whether similar density-dependent socioeconomic gradients emerge in other settings and for other health outcomes and should further investigate the mechanisms through which population density and socioeconomic conditions jointly relate to population health.

## Data Availability

The original contributions presented in the study are included in the article/Supplementary Material, further inquiries can be directed to the corresponding author.
